# Review of Lipid-Lowering Therapy in Women from Reproductive to Postmenopausal Years

**DOI:** 10.31083/j.rcm2305183

**Published:** 2022-05-19

**Authors:** Celeste Witting, Ankita Devareddy, Fatima Rodriguez

**Affiliations:** ^1^Stanford University School of Medicine, Stanford, CA 94305, USA; ^2^Division of Cardiovascular Medicine, Stanford Healthcare, Stanford, CA 94305, USA

**Keywords:** women, cholesterol, hyperlipidemia, lipid lowering therapy, statins, cardiovascular disease

## Abstract

Although cardiovascular disease (CVD) is the leading cause of death in women, cardiovascular risk factors remain underrecognized and undertreated. Hyperlipidemia is one of the leading modifiable risk factors for CVD. Statins are the mainstay of lipid lowering therapy (LLT), with additional agents such as ezetimibe and proprotein convertase subtilisin/kexin type 9 (PCSK9) inhibitors as additive or alternative therapies. Clinical trials have demonstrated that these LLTs are equally efficacious in lipid lowering and cardiovascular risk reduction in women as they are in men. Although the data on statin teratogenicity is evolving, in times of pregnancy or attempted pregnancy, most lipid-lowering agents are generally avoided due to lack of high-quality safety data. This leads to limited treatment options in pregnant women with hyperlipidemia or cardiovascular disease. During the perimenopausal period, the mainstay of lipid management remains consistent with guidelines across all ages. Hormone replacement therapy for cardiovascular risk reduction is not recommended. Future research is warranted to target sex-based disparities in LLT initiation and persistence across the life course.

## 1. Introduction

Cardiovascular disease (CVD) is the leading cause of death for women worldwide [1], yet it remains underrecognized and undertreated. Hyperlipidemia (HLD) is a significant risk factor for CVD and lipid-lowering therapy (LLT) is a cornerstone of CVD treatment and prevention [2]. Despite evidence-based guidelines on management of HLD, 52.3 million American women (40.4%) have total cholesterol (TC) greater than 200 mg/dL [1]. Women are more likely to have high cholesterol than men [1, 3], yet they comprise only 28% of patients in large trials on LLT [4, 5]. Women are also less likely to be treated for high cholesterol; lipid control is seen in 50.5% of women compared to 63.3% of men [3]. Although medical advances have increased lipid management strategies, the decline in cholesterol is more pronounced in men than women [3].

**Fig. 1.**
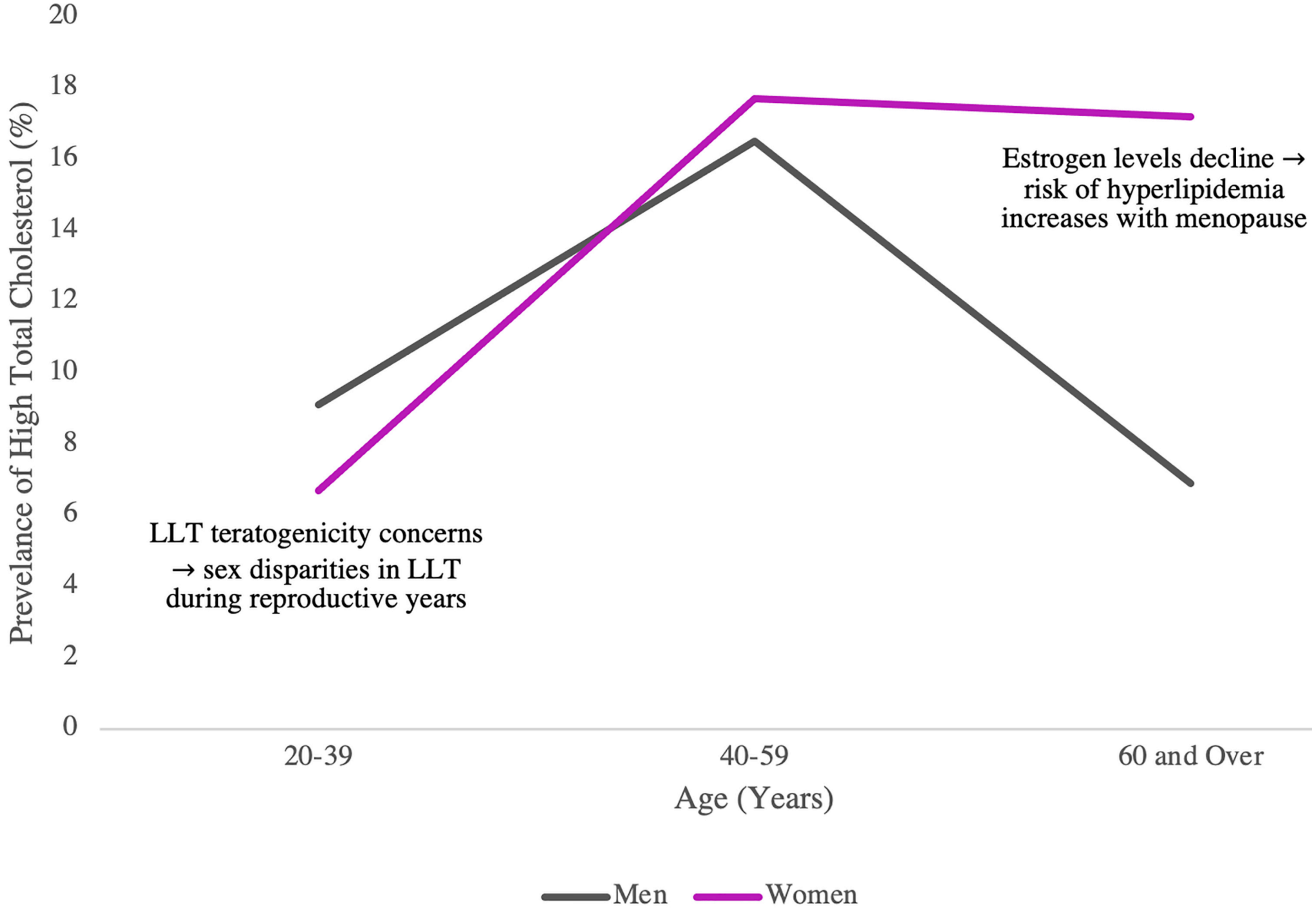
**Hyperlipidemia and LLT in women through the course of life**. LLT, lipid lowering therapy. Prevalence data was reported by the National Health and Nutrition Examination Survey [6]. High total cholesterol was defined as ≥240 mg/dL, and the data reflects United States adults, aged 20 years and older, in 2015–2016.

The purpose of this article is to review current evidence surrounding LLT in women. We will review the efficacy and current state of statin use in women. We will also discuss the use of non-statin lipid lowering therapies such as ezetimibe and PCSK9 inhibitors in women, as well as medications that target triglyceride lowering (i.e., fibrates and omega-3 fatty acids). Finally, we will focus on how lipids and corresponding LLT shift through the lifespan, particularly during times of pregnancy or attempted pregnancy and in the menopausal transition (Fig. 1, Ref. [6]).

It is important to highlight the distinction between sex and gender. Sex is assigned based on anatomy at birth, while gender relates to identity and social factors. Most CVD studies do not distinguish between sex and gender in demographic information, nor do they discuss nonbinary individuals. For this reason, we refer to “men” and “women” and make “sex-based” comparisons, in this review and acknowledge this limitation.

## 2. Statin Efficacy

HMG-CoA reductase inhibitors, or statins, act by inhibiting cholesterol biosynthesis [7], and typically lower low-density lipoprotein-cholesterol (LDL-C) by >50% at the highest intensity and dosing [8]. Statins have proven efficacy in both primary and secondary prevention of major adverse cardiac events (MACE) and improve cardiovascular outcomes in both men and women. Statins have a Class Ia indication for adults aged 40–75 years with diabetes or with 10-year ASCVD (Atherosclerotic Cardiovascular Disease) risk >7.5% [2].

Several landmark statin trials have demonstrated the efficacy of statins for primary prevention of CVD in both men and women. The Justification for the Use of Statins in Primary Prevention: An Intervention Trial Evaluating Rosuvastatin (JUPITER) trial included participants with LDL-C of at least 130 mg/dL without CVD and randomized them to either rosuvastatin (20 mg) or placebo [9]. The trial was stopped early because the statin group had reductions in both MACE and all-cause mortality. This sample was 38.2% women, and there was a similar risk reduction between sexes. Several other large studies have similarly shown the efficacy of statins for primary prevention of CVD in both men and women [10, 11].

Reviews and early meta-analyses suggested that statins may not be as efficacious in women as they are in men for the primary prevention of CVD [12, 13]. This was largely due to the limitation of early statin studies that underrepresented women and were therefore underpowered to analyze efficacy in women; multiple studies and trials have since disproven a sex-based difference in efficacy. In 2015, the Cholesterol Treatment Trialists’ (CTT) Collaboration released a meta-analysis of 27 statin therapy trials for primary prevention, totaling >174,000 participants (27% women) [14]. This analysis showed that risk reduction with statins was similar in men and women for MACE as well as all-cause mortality.

In secondary prevention of CVD, statins have been demonstrated to be equally effective in women as in men. The Treating to New Targets (TNT) trial randomized patients with coronary heart disease (CHD) and LDL-C below 130 mg/dL to either 10 mg or 80 mg of atorvastatin daily [15]. The high-intensity statin group had lower rates of MACE than the low-intensity statin group. Although women were only 19% of this study, there was no statistical interaction of sex with risk reduction. Other studies have similarly supported the benefit of secondary CVD prevention with statins, regardless of sex [16, 17].

## 3. Disparities in Statin Treatment

**Fig. 2.**
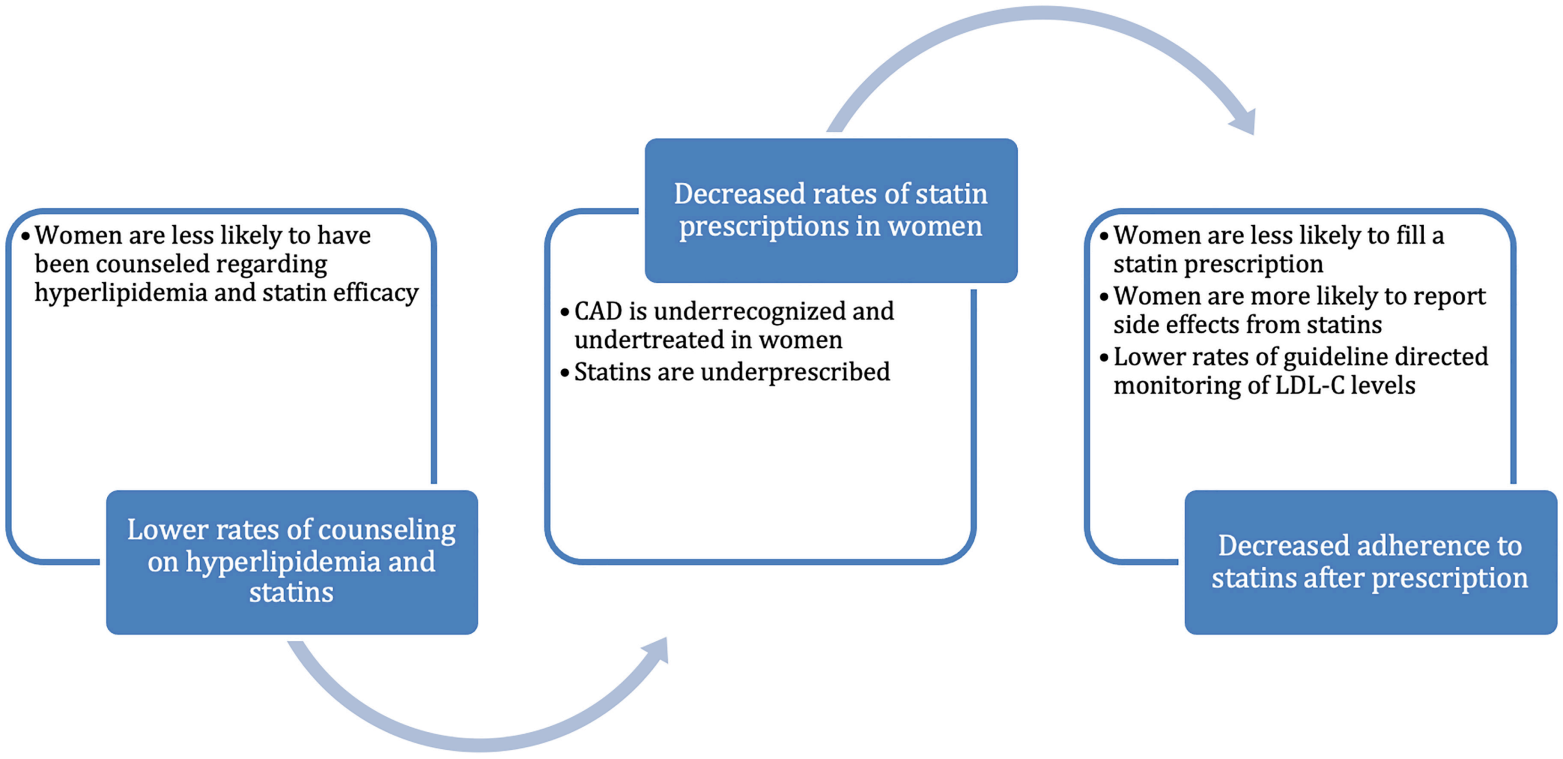
**Causes of decreased statin use and adherence in women**. CAD, coronary artery disease; LDL-C, LDL cholesterol.

Despite the proven efficacy of statins, women are significantly less likely to be treated with statins than men [1, 18, 19]. Sex differences in statin treatment occur at every stage, from counseling to prescription to adherence and monitoring (Fig. 2).

The first step in LLT is counseling patients on its importance. Women are more likely to see doctors regularly than men [20, 21], and continuity of care is generally associated with greater rates of statin adherence [22]. However, women are less likely than men to be informed by their doctor about their risk of heart disease [23], less likely to be offered a statin [23, 24], and less likely to believe that statins are safe and effective [24].

It is thus unsurprising that women are also less likely to be prescribed statins than men. An analysis of the Patient and Provider Assessment of Lipid Management (PALM) Registry and the Department of Veterans Affairs found that, compared to male patients, female patients are less likely to be prescribed a statin at any dose, and even less likely to be prescribed the recommended intensity, as either primary or secondary prevention [24, 25]. Women were also significantly less likely to be treated with statins after hospitalization for a myocardial infarction [26].

Although differences in prescribing explain part of the sex disparity in treatment rates, there is more to the story. If women are prescribed statins, they are less likely to fill their prescription, and have greater rates of discontinuation in the first several months than men [27]. This may be partially explained by underestimation of risk, given that female patients are likely to believe that their cardiovascular risk is lower than that of their male counterparts [28]. Women are also more likely to experience side effects from statins, and to discontinue their statin as a result [23, 24]. Finally, amongst patients who are on statins, women are less likely to receive guideline-directed medication monitoring across all medications, including follow-up lipid panels [29].

The question remains as to why both prescriber and patient attitudes towards statin therapy differ by patient sex. Prescribers are likely influenced by early statin trials that indicated lower efficacy in female patients compared to male patients [12, 13]. However, sex differences in statin efficacy in reducing cardiovascular mortality have now been thoroughly disproven [9, 10, 14, 15, 16, 17].

Muscle side effects are more common in women [12, 13], and this is a common reason for discontinuation. However, it is worth noting that, although myalgias are commonly noted with statins (7–29% of patients) [30], less than half of patients with a history of statin myalgias have recurrence of symptoms with a statin retrial but not placebo [31]. In fact, a quarter of previously intolerant patients have side effects with placebo but not with statin [31]. Continuing statins after an adverse reaction is associated with improved mortality and lower risk of cardiovascular events than alternative methods [32]. It is therefore recommended to continue trying alternative statins or different doses and managing side effects without discontinuation, if able. Given that women are more likely to report myalgias, appreciating the importance of statin re-trial is particularly salient to women.

Overall, the reasons for sex-based disparities in statin therapy are multifactorial and require multiprong strategies that target patient, clinician, and systems barriers. Statins remain the cornerstone of LLT in women, and lower rates of statin treatment are likely a key reason why women have poorer lipid control compared to men [1, 3].

## 4. Non-Statin Therapies: Ezetimibe and PCSK9 Inhibitors

Women who meet a guideline-directed indication for LLT for but who have inadequate LDL-C control on maximally tolerated statins may require an additional lipid-lowering agent (Table 1, Ref. [7]).

**Table 1.** **First and second line therapies to lower low density lipoprotein cholesterol in women**.

Class of Agents	Mechanism	Efficacy in women	*Recommended use	Teratogenicity
Statins	Inhibit hepatic cholesterol biosynthesis	Comparable to men. Lower LDL-C by 50% and decrease CVD.	In adults age 40–75 years, indicated if 10-year ASCVD risk is at least 7.5% (class I).	FDA Pregnancy Category X
Ezetimibe	Prevent cholesterol absorption at the small intestinal brush border	Comparable to men. Lower LDL-C by 13–20% and decrease CVD in high-risk individuals.	In patients with inadequate lipid control on maximally tolerated statin, add ezetimibe (class IIa).	Not studied
PCSK9 Inhibitors	Promote recycling of LDL receptors, thus allowing clearance of LDL from plasma	Comparable to men. Lower LDL-C by ∼60% and decrease CVD risk. Rarely cost-effective.	In patients with inadequate lipid control on maximally tolerated statin and ezetimibe, it is reasonable to add a PCSK9 inhibitor (class Iia).	Not studied

*These recommendations are based on 2019 ACC/AHA guidelines [7]. CVD, cardiovascular disease; LDL-C, low density lipoprotein cholesterol; PCSK9, proprotein convertase subtilisin/kexin type 9.

Ezetimibe is a lipid-lowering agent that acts by blocking cholesterol absorption at the intestinal brush border [33]. For high-risk secondary prevention patients, the multisociety 2018 cholesterol guidelines recommend ezetimibe for whose LDL-C remains above 70 mg/dL despite maximally tolerated statin doses [8].

Ezetimibe is efficacious in lowering LDL-C in both men and women. Compared with placebo, ezetimibe lowers LDL-C by 17% [34], while statins lower LDL-C by >50%, so ideally ezetimibe is added to statin therapy rather than used as a single agent. Ezetimibe and statin therapy together lower LDL-C and raise high-density lipoprotein-cholesterol (HDL-C) more than therapy with a statin alone [35, 36], and this benefit does not vary by sex [35].

Following evidence on the efficacy of ezetimibe for lowering LDL-C, the Improved Reduction of Outcomes: Vytorin Efficacy International Trial (IMPROVE-IT), which comprised 24% women, studied ezetimibe in patients hospitalized following acute coronary syndrome (ACS) [37]. All patients received simvastatin (40 mg) and were randomized to either ezetimibe or placebo. Results showed that the combination therapy group had a lower rate of MACE after 7 years than statin-only therapy (32.7% vs 34.7%, *p *= 0.016) [37, 38]. Compared with men, women had an equal reduction in LDL-C, and a trend towards greater benefit with combination therapy compared to statin-only therapy [39]. When the total number of MACE was considered (rather than the composite), there was an even greater difference, although still not statistically significant (18% vs 6%, *p *= 0.08) [39].

Overall, ezetimibe has proven utility in lipid lowering as well as secondary prevention of ASCVD, in women as much as in men.

Another alternative or additive class of lipid-lowering agents are the proprotein convertase subtilisin/kexin type 9 (PCSK9) inhibitors, evolocumab and alirocumab, which are given as bi-monthly subcutaneous injections. These medications promote recycling of LDL-C receptors on hepatocytes, allowing greater clearance of LDL-C from the plasma [40]. They have been proven to shrink atheromas in patients with angiographic coronary artery disease (CAD) [41]. Multiple clinical trials have shown that PCSK9 inhibitors lower LDL-C by approximately 60% and prevent MACE more than standard therapy without PCSK9 inhibitors [42, 43, 44]. These studies have comprised 24.6–50.5% women, and have shown that efficacy is similar by sex, although one trial did show a more modest LDL-C reduction in women compared to men (53.5% vs 65.5% in men, *p* = 0.0014) [43], and another showed a greater rate of adverse events in women compared to men [44, 45]. Inclisiran is a new PCSK9 inhibitor that was recently FDA approved, given in biannual injections. Inclisiran lowers LDL by approximately 50% in patients on maximally tolerated statins; this was shown in two large trials that comprised 29.4% women, and the LDL-lowering effect was not modified by sex [46].

Given the proven efficacy of both ezetimibe and PCSK9 inhibitors, there has been a question of which agent is best in patients with inadequate lipid control on maximally tolerated statin. Two large trials have both shown that PCSK9 inhibitors reduce LDL-C more than ezetimibe in patients with statin intolerance, for both men and women [31, 47]. However, the annual cost of PCSK9 inhibitors is roughly 75 times the cost of ezetimibe [48]; the cost of PCSK9 inhibitors would need to be lowered in order for them to be cost effective in preventing MACE [49]. The issue of cost effectiveness is particularly salient in treating women, as women’s average full-time income is 82% that of men [50]. Thus, PCSK9 inhibitors are a reasonable additive to statin therapy (or alternative in statin intolerant patients), but ezetimibe is the preferred second-line agent in women as well as in men [8].

## 5. Alternative Therapies for LDL-C Lowering 

Although statins, ezetimibe, and PCSK9 inhibitors are the mainstay of LLT in women, there are alternative therapies that may be applicable for LDL lowering in select patients.

Bile acid sequestrants such as cholestyramine promote modest lowering of LDL-C (9–18%) and TC, raise HDL-C slightly, and may improve cardiovascular outcomes [51, 52]. These effects are similar in men and women [51].

Newer agents may also gain prevalence in the future. Bempedoic acid, which inhibits cholesterol biosynthesis, has been shown to decrease LDL-C by around 17% [53, 54]. In a pooled analysis of four trials on bempedoic acid, it appears that LDL-C reduction was greater in women than in men (–21.2% vs –17.4%, *p* = 0.04) [55].

Another novel agent is evinacumab, which promotes the activity of lipoprotein lipase and endothelial lipase. In patients with refractory HLD, evinacumab reduces LDL-C by >50% [56, 57]. Women were well-represented in these trials (54–62% of patients), but authors did not report whether sex had an interaction with efficacy.

In summary, although statins, ezetimibe, and PCSK9 inhibitors are the mainstay of treatment of HLD in women, other agents may be used as alternatives or additives in select cases. There is no evidence that these agents are less efficacious in women than they are in men.

## 6. Triglyceride Lowering Therapy

The majority of this review focuses on agents that lower LDL-C because of the proven relationship between LDL-C lowering and reduction of ASCVD events. However in some patients, targeting triglycerides (TG) may provide additional benefit. TG levels 175–499 mg/dL are atherogenic, and above 500 mg/dL can precipitate pancreatitis [8]. Women have higher TG than men due to hormonal factors across the lifecourse [58], so management of hypertriglyceridemia (HTG) is particularly relevant in women.

In patients with severe HTG and an estimated 10-year ASCVD risk greater than 7.5%, statin therapy remains first line [8], as statins reduce TG by 10–30% [59]. For patients with isolated HTG or whose TGs remain persistently elevated despite statin therapy, an alternative agent (namely a fibrate or omega-3 fatty acid agent) may be indicated in addition to lifestyle changes [8, 59].

Fibrate therapies such as fenofibrate lower TG by 18–45% [60]. In a systematic review of 18 trials that analyzed the effects of fibrates on cardiovascular outcomes found that fibrates lower the risk of MACE and coronary events, but not stroke, all-cause mortality, or CV mortality [61]. Notably, nearly half of these trials enrolled only men; men were, on average, 82.5% of participants [61]. For this reason, it is difficult to assess the role of fibrate therapy for women with HTG, particularly in terms of CV health.

Omega-3 fatty acids may also be used to lower TG [62], but data on their CV benefit is mixed [59]. The Reduction of Cardiovascular Events with EPA Intervention Trial (REDUCE-IT) showed that, in patients with HLD and concomitant HTG, adding 4 grams of icosapent ethyl, a highly purified eicosapentaenoic acid ethyl ester, to statin therapy reduced the rates of MACE compared to adding mineral oil, and women benefited similarly to men [63]. However, this benefit has not extended to other fish oils [59], with two other studies in addition to REDUCE-IT showing an increased risk of atrial fibrillation [59, 63, 64, 65].

Thus, in patients with HTG, the mainstay of therapy is lifestyle modification, statins, and icosapent ethyl for this with an elevated ASCVD risk. The cardiovascular benefits of alternative methods for TG lowering (including other types of fish oils) has not been documented.

## 7. Sex-Specific Factors Across the Lifecourse

In order to understand how to effectively manage cholesterol in women, it is important to also review how this treatment shifts during two distinct phases of a woman’s life: peri-pregnancy years for those who choose to have children and the menopausal transition. Female endogenous hormones (i.e., estrogens and progestins) impact lipid metabolism, resulting in shifts in lipid levels during these phases.

Estrogen generally improves lipid profiles by acting to increase LDL-C receptors and decrease the production and size of LDL-C, thus reducing circulating LDL-C levels [58, 66, 67]. It also inhibits hepatic lipase and decreases scavenging of HDL-C, thus increasing HDL-C levels [66]. Finally, estrogen decreases Lp (a) through an unknown mechanism and has antioxidant properties [66]. However, it is also thrombogenic (increases prothrombin, decreases antithrombin III), so increases the risk for thromboembolic events [68].

By contrast, progestins tend to have a detrimental effect on lipid profiles, by increasing LDL-C and decreasing HDL-C [66]. They thus tend to counteract the effects of the effects of estrogen on cholesterol levels. For example, although estrogen inhibits intimal thickening in arteries, progesterone dose-dependently inhibits this beneficial effect [69].

This background is helpful to understand the physiologic changes in lipid profiles that occur during pregnancy (section 8) and menopause (section 9).

## 8. LLT in Pregnancy

During pregnancy, as estrogen and progesterone steadily rise, so too does TC (both LDL-C and TG) [70]. Cholesterol levels decrease during the first six weeks of pregnancy, but then increase throughout gestation and peak at delivery; TC increases from an average of 164.4 to 238.6 mg/dL [71]. Reference ranges for normal lipid levels should thus be adjusted during pregnancy [72].

Despite rising lipids during pregnancy, women who are pregnant or desiring pregnancy are not a large proportion of all patients requiring LLT. Lifetime risk of ASCVD is higher in men than in women, and women tend to develop HLD later in life than men [73]. However, approximately 27% of ASCVD-free young adults have LDL-C levels of at least 130 mg/dL, and although treatment is currently indicated for only a minority of these patients with current guidelines, a recent analysis showed that statin therapy for young adults with LDL-C ≥130 mg/dL prevents ASCVD and increases quality-adjusted life years [74]. Additionally, as women are having children later in life [75], the frequency of treating HLD in pregnancy is increasing. HLD in pregnancy is also associated with maternal morbidity, mortality, and preterm delivery, and women who deliver preterm are at a higher risk for cardiovascular disease later in life [76]. It is thus important to be familiar with the teratogenicity of lipid-lowering agents in order to optimize lipid management in women of reproductive age.

Statins have a Pregnancy Category of X from the United States Food and Drug Administration (FDA) and are classically contraindicated in pregnancy as well as while breastfeeding [77]. The current ACC/AHA guidelines recommend that women of childbearing age who take statins should be on reliable contraception, and if they want to pursue pregnancy, should stop the statin 1–2 months before pregnancy is attempted [8, 78]. The results of this is that women with HLD, including women with familial hypercholesterolemia, lose years of statin treatment during attempted reproduction [79].

However, much of the data on statin teratogenicity is based on animal studies where subjects were given higher doses of statins than are used in clinical practice [80]. Since statins were deemed unsafe in pregnancy, there has been understandably little clinical data on fetal outcomes because few pregnant women are treated with statins. An array of smaller observational studies have shown mixed results on the effects of statins on fetal health. Although some studies show an association between statin use and fetal central nervous system and limb anomalies, systematic reviews and meta-analyses of later and larger studies have not shown a relationship between statins and congenital anomalies when controlling for risk factors [81, 82, 83]. These results suggest that the association between statins and poor fetal outcomes is confounded, at least in part, by maternal comorbidities such as diabetes and obesity, which themselves contribute risk to fetal health [81, 82, 83].

There is growing interest in reconsidering the safety of statins in pregnancy and lactation. In 2016, the FDA approved a small trial which randomized twenty pregnant women (at 12–16 weeks gestation) at high risk of pre-eclampsia to either pravastatin (10 mg) or placebo [84]. The results showed no difference in congenital anomalies or fetal outcomes, but the group on pravastatin had decreased rates of pre-eclampsia. Additionally, pravastatin lowered maternal cholesterol without impacting umbilical cord cholesterol, indicating that it is possible for therapies to impact maternal lipid profiles without impacting fetal development.

In light of this evidence, the safety of statins in pregnancy is an evolving topic, and larger studies are needed as appropriate. New guidelines from the FDA eased the severity of their recommendation against statins in pregnancy to allow providers and patients to make decisions on an individual basis in high-risk circumstances [77]. If the decision is made to treat a pregnant woman with a statin, it is best to choose a hydrophilic option (e.g., pravastatin) because these are less likely to affect the embryo than lipophilic formulations [81]. In sum, statin medications are best avoided in pregnancy in most circumstances, with an awareness that there will likely be more evidence on this topic in coming years.

Given that statins are generally contraindicated in pregnancy and lactation, there would ideally be efficacious and safe alternatives for LDL-C lowering in women. However, both ezetimibe and PCSK9 inhibitors have not been studied in pregnancy or lactation to determine their effects [78], although there is an ongoing trial evaluating the teratogenicity of evolocumab [85].

Safe options for LDL-C lowering in pregnancy include bile acid sequestrants, which are not systemically absorbed and thus are deemed to be safe in pregnancy and during breastfeeding. However, it is important to counsel patients that these medications interfere with the absorption of fat-soluble vitamins that are important for the health of both patient and fetus [86]. Vitamins should thus be taken at separate times of the day from their medication to promote absorption, and it may be helpful to monitor maternal INR in patients taking bile acid sequestrants to ensure there is adequate vitamin K absorption to prevent maternal and fetal hemorrhages [86].

Although LDL-C and TC rise during pregnancy, the greatest increase is in TG, which normally double or triple throughout pregnancy [70]. This change is normal and physiologic, but excessively high TG levels are associated with increased risks of macrosomia and preterm birth [87]. For management, fibrates have FDA Pregnancy Category C are not recommended [70]. Omega-3 fatty acids are safe in pregnancy and recommended in some patients who have low omega-3 intake; in these patients; omega-3 supplementation may reduce risks of early preterm birth [88].

Currently the treatment for HLD in pregnant women is primarily nonpharmacologic, by emphasizing a healthy lifestyle and low-fat low-cholesterol diet with exercise [70]. Due to limited pharmacologic options with proven safety, high-risk pregnant women, such as those with homozygous familial hyperlipidemias, may be treated with therapeutic plasma exchange or LDL-C apheresis [89]. Overall, further research on teratogenicity of LLT is required to appropriately treat a growing population of pregnant patients with HLD.

## 9. LLT in Menopause

Menopause represents another distinct shift in a woman’s hormonal balance. When women enter menopause, they have decreased estrogen production from the ovaries, and thus worsening of their lipid profiles; TC, LDL-C and TGs all increase. The odds ratio for having an LDL-C level of at least 130 mg/dL is 2.1 for early postmenopausal women compared to premenopausal women [90]. Although this change may in theory be explained by age, surgical ovary removal has also been shown to increase LDL-C levels [91], and TC and LDL-C substantially increase in the year around the final menstrual period [92], which helps distinguish this increase from solely age-related changes [92, 93, 94].

Given the detrimental effects of menopause on lipids, it may seem intuitive that hormone replacement therapy (HRT) would promote cardiovascular health. Early observational research showed improved lipid profiles and lower CV risk with HRT. Supplemental estrogen, with or without supplemental progestin, was shown to decrease both TC and LDL-C in multiple studies [95, 96, 97]. Additionally, in women undergoing angiograms, lower rates of CAD were seen in patients taking supplemental estrogen than women without HRT [98], although this is not necessarily causative and there are several potential confounders to this association. Based on this evidence of the impact of HRT on lipid profiles, HRT was recommended as first-line therapy for HLD in postmenopausal women in 1993 [99].

Although it is helpful to understand former use of HRT for CV risk reduction from a historical perspective, in the decade that followed this recommendation, multiple studies provided contrary evidence that altered this recommendation. First, a small crossover study where women received both a statin and combined HRT sequentially showed that statin therapy was more efficacious in lowering both TC and LDL-C than HRT [100]. Then, the Heart and Estrogen/Progestin Replacement Study (HERS) trial randomized 2763 postmenopausal women with CAD to either combined HRT or placebo [101]. The results showed no significant difference in myocardial infarction or cardiovascular death with HRT, but the treatment group had an increased risk of thrombotic events. The Women’s Health Initiative (WHI) trial randomized 16,608 postmenopausal women to combined HRT or placebo and found that treatment was associated with higher risks of stroke, pulmonary embolism, and cardiovascular death [102]. This trial was stopped early due to an increased risk of breast cancer in the HRT group. In the WHI trial, the increased CVD risk was driven by the progestin component, as increased CVD risk was not seen when women received estrogen alone [103]; the risk of thromboembolic events is also higher with combined therapy than with estrogen alone [104]. However, unopposed estrogen increases the risk of endometrial cancer in women with intact uteri [104].

Combining this evidence, a review of all studies related to HRT and cardiovascular health found no benefit of HRT for primary or secondary prevention of cardiovascular events, with a trend towards harm [58]. The FDA now has a black box warning to estrogen preparations, specifying that these agents should not be used for cardiovascular health [105]. HRT should only be used in women with moderate to severe menopausal symptoms and should not be taken for more than three years [105]. In postmenopausal women, statins are still first line therapy, followed by ezetimibe and PCSK9 inhibitors.

## 10. Conclusions

Compared with men, women remain undertreated with guideline-directed LLT across the spectrum of cardiovascular risk. Women should be treated for HLD following standard guidelines, with statins as first line, as the major lipid-lowering agents have comparable efficacy in women as they do in men. The exception to this is during times of attempted pregnancy since statins, ezetimibe, and PCSK9 inhibitors are not proven to be safe in pregnancy. Finally, in the menopausal transition, HRT should not be used for CVD risk reduction in menopausal patients despite early evidence that it may be beneficial; the mainstay remains statin therapy. Future research is warranted to understand how to expand treatment options for HLD across the life course for women, including pregnancy and during the menopausal transition. Guideline-directed prescriptions and adherence to prescribed LLT should be monitored regularly.
